# Activation of Exchange Protein Activated by cAMP in the Rat Basolateral Amygdala Impairs Reconsolidation of a Memory Associated with Self-Administered Cocaine

**DOI:** 10.1371/journal.pone.0107359

**Published:** 2014-09-26

**Authors:** Xun Wan, Mary M. Torregrossa, Hayde Sanchez, Angus C. Nairn, Jane R. Taylor

**Affiliations:** Department of Psychiatry, Yale University School of Medicine, New Haven, Connecticut, United States of America; Sapienza University of Rome, Italy

## Abstract

The intracellular mechanisms underlying memory reconsolidation critically involve cAMP signaling. These events were originally attributed to PKA activation by cAMP, but the identification of Exchange Protein Activated by cAMP (Epac), as a distinct mediator of cAMP signaling, suggests that cAMP-regulated processes that subserve memory reconsolidation are more complex. Here we investigated how *activation* of Epac with 8-pCPT-cAMP (8-CPT) impacts reconsolidation of a memory that had been associated with cocaine self-administration. Rats were trained to lever press for cocaine on an FR-1 schedule, in which each cocaine delivery was paired with a tone+light cue. Lever pressing was then extinguished in the absence of cue presentations and cocaine delivery. Following the last day of extinction, rats were put in a novel context, in which the conditioned cue was presented to reactivate the cocaine-associated memory. Immediate bilateral infusions of 8-CPT into the basolateral amygdala (BLA) following reactivation disrupted subsequent cue-induced reinstatement in a dose-dependent manner, and modestly reduced responding for conditioned reinforcement. When 8-CPT infusions were delayed for 3 hours after the cue reactivation session or were given after a cue extinction session, no effect on cue-induced reinstatement was observed. Co-administration of 8-CPT and the PKA activator 6-Bnz-cAMP (10 nmol/side) rescued memory reconsolidation while 6-Bnz alone had no effect, suggesting an antagonizing interaction between the two cAMP signaling substrates. Taken together, these studies suggest that activation of Epac represents a parallel cAMP-dependent pathway that can inhibit reconsolidation of cocaine-cue memories and reduce the ability of the cue to produce reinstatement of cocaine-seeking behavior.

## Introduction

Vulnerability to stimulus-induced drug craving is a major obstacle for the treatment of compulsive drug seeking and prevention of relapse. Exposure therapy based on extinction procedures alone has generally been unsuccessful, partially because the effects of extinction are highly context dependent and not long lasting [1]–[5]. Based on the notion that memories (such as those involving drug-cue associations) go through restabilization following retrieval (known as “reconsolidation”), it has been argued that blockade of memory reconsolidation may be an effective strategy in the treatment of drug addiction, as this would weaken the strength of drug memories to reduce relapse. Moreover, unlike extinction, disrupting reconsolidation can produce enduring effects that are context independent [3], [6], [7]. Thus, identifying mechanisms for disrupting the reconsolidation of memories associating drugs with environmental cues has the potential to improve the treatment of addiction.

The amygdala has been extensively studied for its role in associative learning, whereby an initially neutral stimulus becomes associated with appetitive or aversive events upon repeated pairings [8], [9]. Inactivation of the basolateral amygdala (BLA) impairs stimulus-induced reinstatement to cocaine seeking in animal models [10], [11]. In addition, cocaine-associated stimuli elicit simultaneous drug craving and robust amygdala activation in abstinent cocaine users [12]–[14]. A handful of studies have begun to specifically investigate how the BLA is involved in memory reconsolidation for drug-associated stimuli. For example, during or shortly following retrieval of a drug-cue memory, systemic and intra-amygdala pharmacological manipulations including NMDA receptor antagonism by MK-801 [15], [16], or by D-APV [17] reduces subsequent performance associated with that cue. BLA knockdown of the immediate early gene *zif268* before reactivation of a cocaine-associated memory also reduces the ability of that CS to serve as a conditioned reinforcer in the acquisition of a new instrumental task [18] or reinstate cocaine-seeking behavior [19].

In addition, we have previously demonstrated that the cAMP-dependent protein kinase, protein kinase A (PKA) within the BLA is required for reconsolidation of both a fear- or a cocaine-associated memory [20], [21], consistent with the well established role of cAMP signaling through PKA in the formation of long-term memories. The mechanism by which cAMP-induced activation of PKA induces memory formation is likely through the phosphorylation of multiple downstream substrates, including receptors, intracellular signaling molecules and transcription factors [22]. However, cAMP does not only signal through PKA, but also activates the exchange protein activated by cAMP (Epac). Epac proteins (Epac1 and Epac2) link cAMP signaling to the activation of small Rap1 GTPases [23], [24]. Epac2 is enriched in brain and expressed in multiple regions involved in memory functions such as hippocampus, amygdala and prefrontal cortex [24]. However, little is known regarding the role of Epac in memory and its involvement in reconsolidation has not been shown. Here, we demonstrate that *activation* of BLA Epac following retrieval of drug-cue memories impairs reconsolidation and consequently reduces the efficacy of cocaine-associated cues to reinstate drug-seeking behavior.

## Materials and Methods

### Subjects

The subjects were 118 experimentally naïve male Sprague Dawley rats, purchased from Charles River Laboratories (300–350 g of weight at the start of the experiment). A total of 16 rats were ultimately removed from the study because of failure to meet acquisition/extinction criteria or due to improper cannula placement. Upon arrival, rats were housed in pairs, given *ad libitum* access to food and water, and allowed to acclimate for at least 7 d before surgery. Following surgery, rats were individually housed. Food restriction was imposed after recovery to maintain the body weight at 90% of the free-feeding level for the duration of the experiment. The animal room was temperature and humidity controlled and maintained on a 12/12 h light/dark cycle (lights on at 7∶00 am). All experiments were conducted during the light phase of the rat cycle.

### Ethics Statement

All procedures were conducted in accordance with the policies of the Yale University Institutional Animal Care and Use Committee under an approved protocol and conformed to National Institutes of Health Guidelines on the Care and Use of Laboratory Animals.

### Surgery

Rats were anesthetized with a mixture of 87.5-mg/kg ketamine and 5-mg/kg xylazine (i.p.) and were given 5 ml of Lactated Ringers solution (s.c.). Rats were implanted with a chronic indwelling catheter (Camcaths, Cambridge, U.K.) into the right jugular vein and a bilateral guide cannula (22 gauge; Plastics One) targeting the BLA (AP −2.5 mm, ML ±4.8 mm, DV −7.6 mm relative to bregma). The patency of the intravenous catheter was maintained by an infusion of a 0.1 ml of heparinized saline (30 USP heparin/saline; Hospira) every 2 d. The intracranial cannulae were kept patent by the insertion of obturators that were replaced daily. Rats were allowed to recover for a week before the start of the training.

### Behavioral Procedures

#### Acquisition of cocaine self-administration

Rats were trained in sound-attenuated operant conditioning chambers (Med Associates). The operant box was equipped with two retractable levers (positioned on either side of a central magazine on the right wall of the operant chamber), a stimulus light located above the active lever, a tone generator, and a fan that provided background noise (65 dB). Rats underwent cocaine self-administration for 12 d in daily 1 h sessions under a fixed-ratio one (FR1) schedule of reinforcement on the active lever. Inactive lever responses were recorded but did not result in the presentation of any stimuli or infusions. Each infusion of cocaine (1.0 mg/kg) was paired with a 10 s light/tone (75–78 dB) CS. Rats had a 10 s timeout between infusions. Rats were removed from the chambers upon termination of the session. Acquisition of self-administration typically required 7 days of training.

#### Extinction of lever pressing

Following acquisition of cocaine self-administration, lever pressing was extinguished for 8 d. During the 1 h daily extinction sessions, both levers were available but responses on either lever had no programmed consequences. Rats were never exposed to the cocaine-paired CS during the extinction sessions.

#### Cue Reactivation

Following the last extinction day, rats underwent stimulus reactivation. Only rats that fulfilled the criteria for acquisition (≥8 infusions averaged over each of the last 3 consecutive self-administration sessions for each individual animal) and extinction (<25 active lever responses) were reactivated. The reactivation session took place in a novel context (different lighting, flooring, novel odor) and three presentations of the 10 s light/tone stimulus were delivered with an inter-stimulus interval of 1 min. The rats had no access to levers during this session. Immediately following the reactivation session (experiments 1, 2 and 4), rats underwent a bilateral infusion into the BLA. Delayed-reactivation controls received infusions 3 h after the reactivation session (experiment 3). Rats were returned to their home cages in the animal colony following the infusion procedure.

#### Cue Extinction

In experiment 5, rather than exposing rats to a cue reactivation session, they underwent a cue extinction procedure as previously published [25]. All parameters were the same as those described above for cue reactivation. The rats were placed in a novel context that was a different size and shape with bar instead of grid floors and an almond odor added to the box. Rats received 60 presentations of the light/tone stimulus on each of 2 daily, 30 min sessions. We have previously found that 120 total cue presentations is sufficient to produce extinction of cue-motivated behavior when rats are tested in the same context as training, but to result in renewal of cue-motivated behavior when cue extinction training and testing occur in different contexts [25]. Rats received bilateral infusions into the BLA immediately following both of the cue extinction sessions.

#### Cue-induced reinstatement testing

Twenty-four hours after the reactivation/infusion or last extinction session, rats were given a cue-induced reinstatement test. During this session, rats were returned to the self-administration context and responding on both levers was recorded for 1 h. Active lever responding resulted in presentation of the light/tone CS but no cocaine infusion. Inactive lever responding had no programmed consequences.

#### Acquisition of a New Response (conditioned reinforcement testing)

In experiments 2 and 5 responding on a conditioned reinforcement test was assessed on the day following the cue-induced reinstatement test. The test of conditioned reinforcement determines to what degree a conditioned cue (in this case a cue associated with cocaine) has developed reinforcing properties by asking how well an animal acquires a new response that is reinforced solely by the cue [26], [27]. Rats were placed in the same boxes used for cue reactivation/extinction for a 1 hr session. The first nose poke on the active nose poke produced a 10 sec presentation of the light/tone stimulus. Responding on an inactive nose poke produced no programmed consequences, but they were recorded following the first response on the active nose poke. Inactive nose pokes were only recorded after an active response because the difference between active and inactive responses indicates that the animal is responding primarily for the reinforcing properties of the cue and this excludes potential initial side biases that could occur before the animal has learned he can respond for reinforcement.

### Drug Infusions

Infusions were performed using a syringe pump with 10 µl Hamilton syringes connected to injection cannula (28 gauge; Plastics One) via polyethylene tubing. Rats received a bilateral infusion (0.25 µl/min, for 2 min) into the BLA [20] and the injectors remained in place for 2 min following the infusion to allow for diffusion of the solution. In experiments 1, 2, 3, and 5, the experimental groups received infusions of the Epac-specific agonist [28] (8-(4-chlorophenylthio)-2′-O-methyladenosine-3′, 5′-cyclic monophosphate (8-CPT, 7.5 or 10 nmol per side, Sigma-Aldrich). In experiment 4a, the experimental group received infusions of a mixture of 8-CPT (10 nmol per side) and the PKA-specific activator 6-Bnz-cAMP (10 nmol per side) [29]. In experiment 4b, the experimental group received infusions of 6-Bnz alone. All controls received an equal volume infusion of vehicle, which was 1× PBS for all drugs.

### Histology

At the end of each experiment, rats were deeply anesthetized (90 mg/kg sodium pentobarbital, i.p.) and infused with thionin (1%; Sigma-Aldrich) into the BLA at the same volume and rate used for experimental drug infusion to subsequently visualize the location of the injector tip. The injectors remained in place for 2 min following the infusion. Animals were euthanized and brains were stored in 4% paraformaldehyde overnight and then transferred to 30% sucrose. The brains were sliced in 50 µm coronal sections using a cryostat at approximately −16°C. Every fourth slice was mounted on a microscope slide. Verification of infusion site was based on Paxinos and Watson [30], and the location was plotted on the coronal plate that most closely corresponded to its anterior–posterior position.

### Statistical Analysis

Acquisition of cocaine self-administration was analyzed using repeated-measures ANOVAs (rm-ANOVAs, IBM SPSS or GraphPad Prism) across days for the following measures: number of infusions, total number of active lever responses, and total number of inactive lever responses. Extinction of lever pressing was analyzed using rm-ANOVAs across days for the following measures: total number of active lever responses and total number of inactive lever responses. Following the acquisition and extinction phrases, the groups were split into the to-be-infused conditions by a “matching” procedure where animals are randomly assigned to treatment groups based on matched levels of behavioral responses and infusions during training and lever extinction. Thus, treatment groups did not significantly differ from each other on any behavioral measures during training prior to histological analysis. Cue-induced reinstatement tests were analyzed using rm-ANOVAs across the last extinction day and the reinstatement test for the following measures: total number of active lever responses and total number of inactive lever responses. All significant rm-ANOVAs were further analyzed with Bonferroni *post hoc* tests (*p*<0.05).

## Results

### Experiment 1: Immediate post-reactivation intra-BLA Epac activation reduces subsequent cue-induced reinstatement

**Figure 1:**
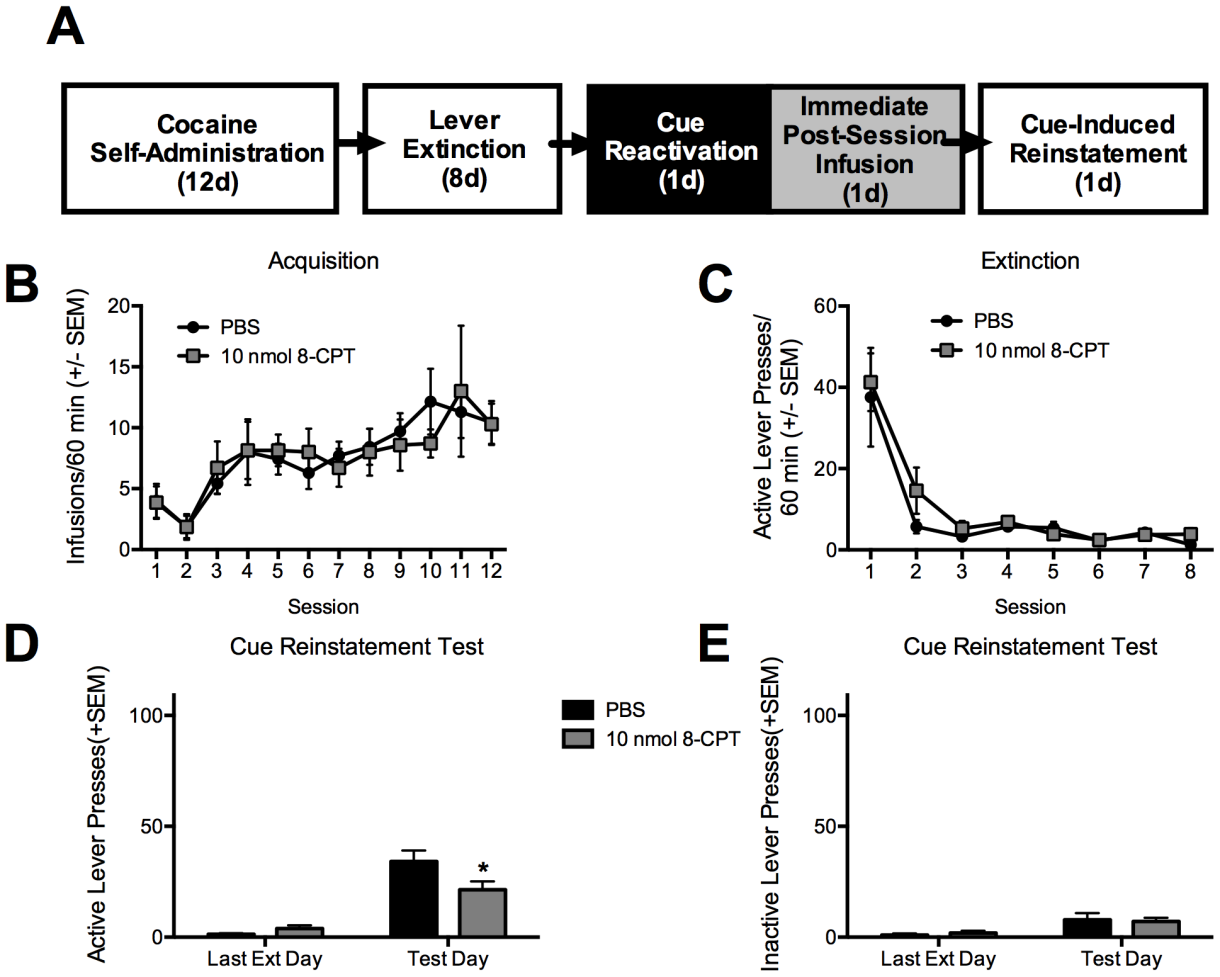
Activation of Epac by 8-CPT cocaine cue memory reactivation selectively reduces subsequent cue-induced reinstatement. A. Schematic representation of the experimental procedure and the timeline. Black and white boxes are used to represent different contexts. B. Total number of cocaine infusions across the 12 acquisition sessions of cocaine self-administration. C. Total number of active lever presses across the 8 extinction sessions. D. Total number of active lever presses across the last extinction day and the cue-induced reinstatement test. E. Total number of inactive lever presses during tests shown in D. All data are represented as the mean +SEM, N = 7/group and *p<0.05.

Both groups of rats acquired cocaine self-administration over the training days and the acquisition was not different between the groups, measured by the number of cocaine infusions (Fig. 1B), active lever presses, or inactive lever presses (all p values >0.05). During the extinction phase, both groups extinguished the lever responses across the 8 sessions and no significant difference was found between the groups, measured by active lever presses or inactive lever presses (Fig. 1C; all p values >0.05). Across the last extinction day and the cue-induced reinstatement test, there was a significant session X group interaction (F_(1,12)_ = 7.92, p<0.05) in active lever presses (Fig. 1D). Further assessments indicated that during the cue reinstatement test the 8-CPT infused group significantly reduced active lever responses compared to vehicle control (Bonferroni test, p<0.05) while the inactive lever responses did not differ between the groups (Fig. 1E). Therefore, activation of Epac immediately following reactivation appears to disrupt subsequent memory reconsolidation, thus reducing the efficacy of the drug cue to reinstate drug-seeking behavior.

### Experiment 2: Dose effect of post-reactivation intra-BLA 8-CPT on cue-induced reinstatement and acquisition of a new response

**Figure 2:**
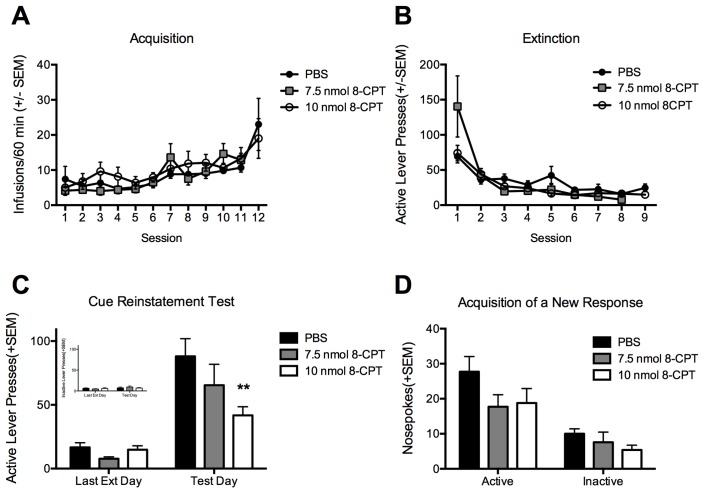
Activation of Epac by 8-CPT reduces cue-induced reinstatement in a dose-dependent manner while moderately reducing conditioned reinforcement. A. Total number of cocaine infusions across the 12 acquisition sessions of cocaine self-administration. B. Total number of active lever presses on each day of extinction training. C. Total number of active lever presses on the last day of extinction compared to the cue-induced reinstatement test. The total number of inactive presses are in the inset figure and did not differ between groups. D. Total number of active and inactive nosepokes on the acquisition of a new response test for conditioned reinforcement. All data are represented as the mean +SEM, PBS N = 12, 7.5 8CPT N = 7, 10 8-CPT N = 12, and **p<0.01.

In order to replicate the above finding, we conducted a dose response analysis of the effect of 8-CPT on both cue-induced reinstatement and on the acquisition of a new response for conditioned reinforcement to assess if the cue memory was more generally disrupted (see [21]). There was no difference between the groups on either the number of cocaine infusions self-administered during training (Fig. 2A) or on the number of lever presses (active or inactive) made during self-administration or extinction (Fig. 2B; all p’s>0.05). Analysis of active lever responses on the cue-induced reinstatement test by two-way ANOVA revealed a significant effect of time [F_(1,28)_ = 49.15, p<0.001], a significant effect of treatment group [F_(2,28)_ = 4.26, p = 0.024], and a significant interaction [F_(2,28)_ = 3.83, p = 0.034]. Post-hoc analysis indicated that the group treated with 10 nmol 8-CPT had a significant reduction in cue-induced reinstatement relative to controls, while the 7.5 nmol had a non-significant reduction in reinstatement (Fig. 2C). There was no difference between groups in inactive lever responding during the cue-induced reinstatement test (Fig. 2C inset). In addition, 24 hours following the cue-induced reinstatement test, the animals were tested in the memory reactivation context for their propensity to acquire a new response (nose poking) solely reinforced by the cocaine-conditioned cue to assess whether the cue retained conditioned rewarding effects after 8-CPT infusion. Two-Way analysis of variance indicated a significant effect of nose poke (active vs. inactive) [F_(1,28)_ = 35.44, p<0.001], but no effect of treatment group or interaction (p’s >0.05). However, because it appeared that both the 7.5 and 10 nmol doses of 8-CPT reduced active responses, but were not significantly different from each other, we combined those two groups and compared to controls using a t-test, and found that both groups combined had responded significantly less than controls for conditioned reinforcement (p = 0.037). These data suggest that while 8-CPT does not prevent acquisition of a new response there is a tendency for 8-CPT to reduce the conditioned reinforcing properties of the cue in a non-dose dependent manner (Fig. 2D).

### Experiment 3: Delayed intra-BLA Epac activation has no effect on subsequent cue-induced reinstatement

**Figure 3:**
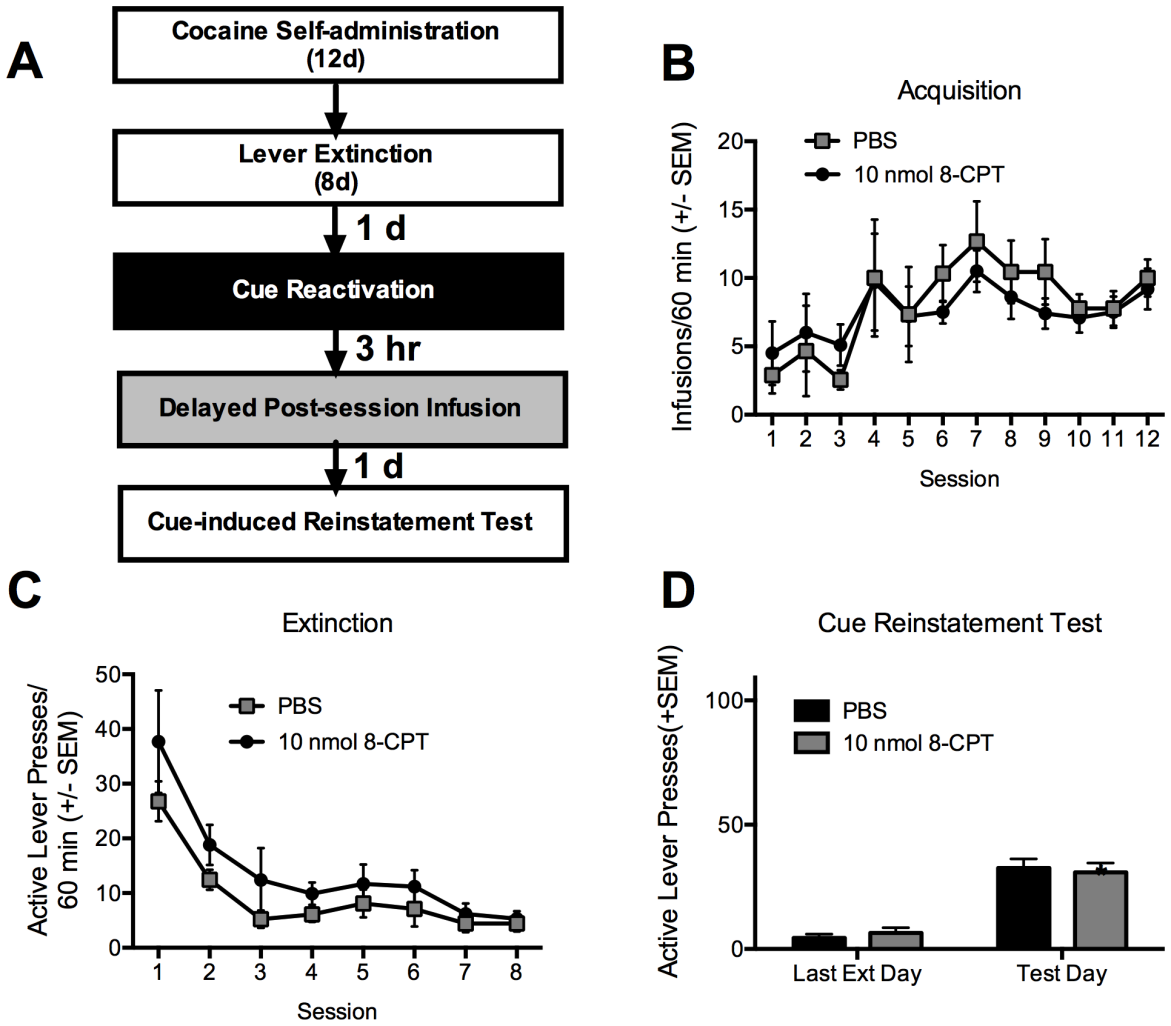
Delayed infusion of 8-CPT after memory reactivation does not alter cocaine cue memory reconsolidation. A. Schematic representation of the experimental procedure and the timeline. Black and white boxes are used to represent different contexts. B. Total number of cocaine infusions across the 12 acquisition sessions of cocaine self-administration. C. Total number of active lever presses across the 8 extinction sessions. There was a significant (p<0.05) main effect of treatment with the 8-CPT group responding more overall than the control group. D. Total number of active lever presses across the last extinction day and the cue-induced reinstatement test. All data are represented as the mean +SEM, N = 9/group.

Again, both groups of rats acquired cocaine self-administration across the training phase and the acquisition did not differ between the groups, according to the number of cocaine infusions (Fig. 3B), active lever presses or inactive lever presses (all p values >0.05). The groups did somewhat differ in their extinction performance with a main effect of treatment (F_(1,136)_ = 6.625, p = 0.011). There was the expected main effect of time indicating that the animals did extinguish, but no interaction effect, indicating that both groups extinguished at the same rate, but that the 8-CPT group started at a higher baseline (Fig. 3C). When 8-CPT or vehicle was infused 3 hrs after the reactivation session, no significant difference was found during the cue-induced reinstatement testing session between the groups (Fig. 3D, session X group interaction: F_(1,16)_ = 0.64, p>0.05; group: F_(1,16)_ = 0.002, p>0.05). While extinction responding can predict future reinstatement, we feel that this is unlikely in this case as the differences between the groups were so small on the reinstatement day. In line with Experiment 1, these results suggest that the effect of Epac activation by 8-CPT is restricted to a temporal window following memory reactivation (i.e., within <3hrs).

### Experiment 4: Disruptions on cue-induced reinstatement caused by Epac activation are blocked by simultaneous activation of PKA while PKA activation alone has no effect

**Figure 4:**
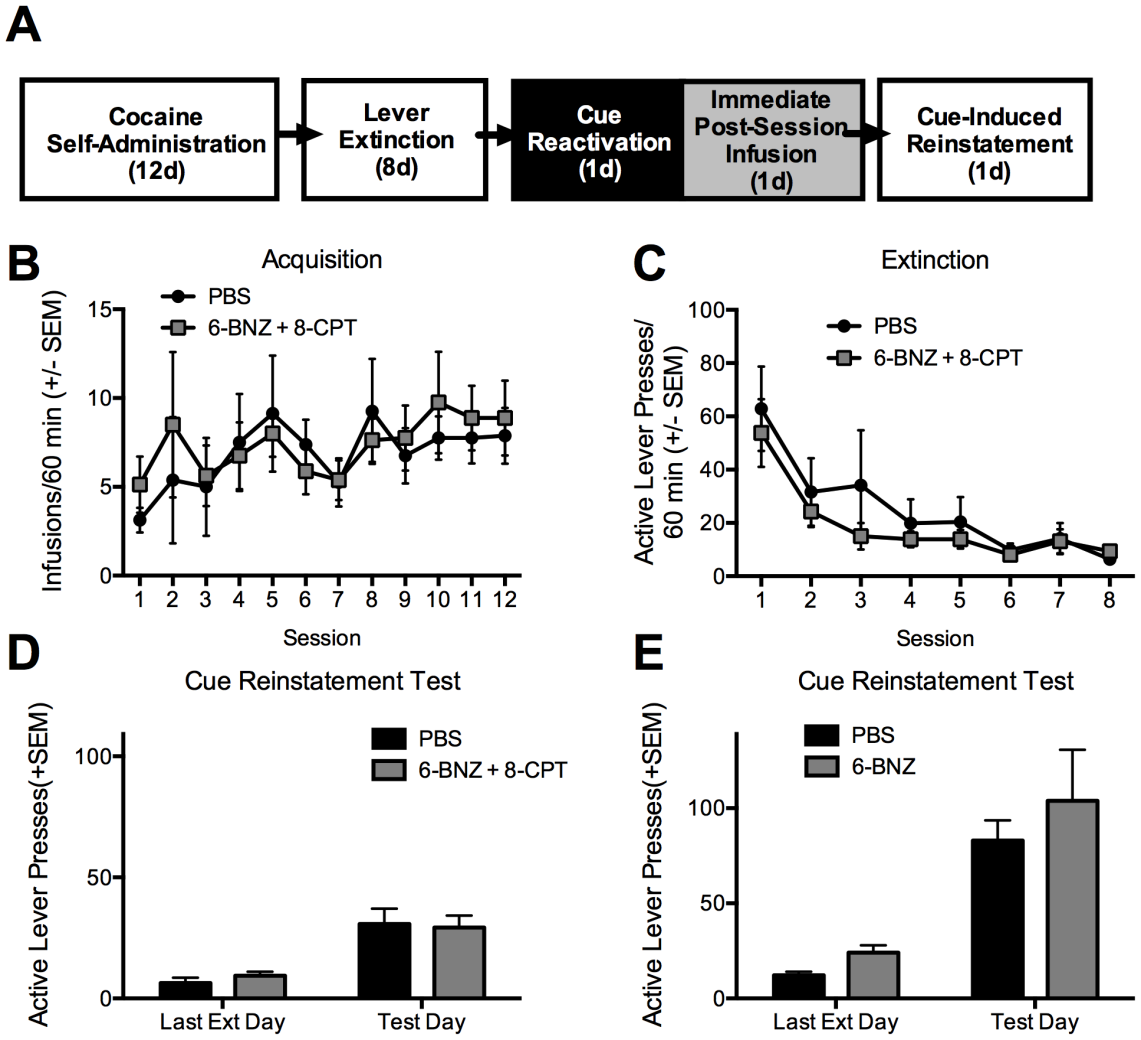
Activation of PKA by 6-BNZ inhibits the reconsolidation disruptive effects of 8-CPT. A. Schematic representation of the experimental procedure and the timeline. Black and white boxes are used to represent different contexts. B. Total number of cocaine infusions across the 12 acquisition sessions of cocaine self-administration. C. Total number of active lever presses across the 8 extinction sessions. D. Total number of active lever presses across the last extinction day and the cue-induced reinstatement test (experiment 4a), N = 8/group. E. Total number of active lever presses across the last extinction day and the cue-induced reinstatement test (experiment 4b), PBS N = 5, 6-BNZ N = 7. All data are represented as the mean +SEM.

In experiment 4a, we tested how simultaneous activation of PKA and Epac would affect cue-induced reinstatement. Both groups of rats acquired cocaine self-administration across the training sessions and the acquisition was not different between the groups, according to the number of cocaine infusions (Fig. 4B), active lever presses or inactive lever presses (all p values >0.05). The groups did not differ in their extinction performance (Fig. 4*C*, p>0.05). When a mixed solution of 8-CPT and 6-Bnz was infused immediately after the reactivation session, there was no significant difference between this treatment and a vehicle control group in active lever presses across the last extinction day and the cue reinstatement testing session (Fig. 4D, session X group interaction: F_(1,14)_ = 0.15, p>0.05; group: F_(1,14)_ = 1.55, p>0.05). In experiment 4b, we tested if PKA activation by 6-Bnz alone would affect cue-induced reinstatement. The groups did not differ in either acquisition or extinction performance. When 6-Bnz or vehicle was infused immediately after the reactivation session, no significant difference was found between the groups in active lever presses across the last extinction day and the cue reinstatement test (Fig. 4E, all p values >0.05). These results suggest that the disruptive effects of Epac activation by 8-CPT were neutralized by simultaneous activation of PKA while PKA activation alone was not sufficient to enhance memory reconsolidation.

### Experiment 5: Epac activation had no effect on Pavlovian extinction or renewal of cocaine-seeking

**Figure 5:**
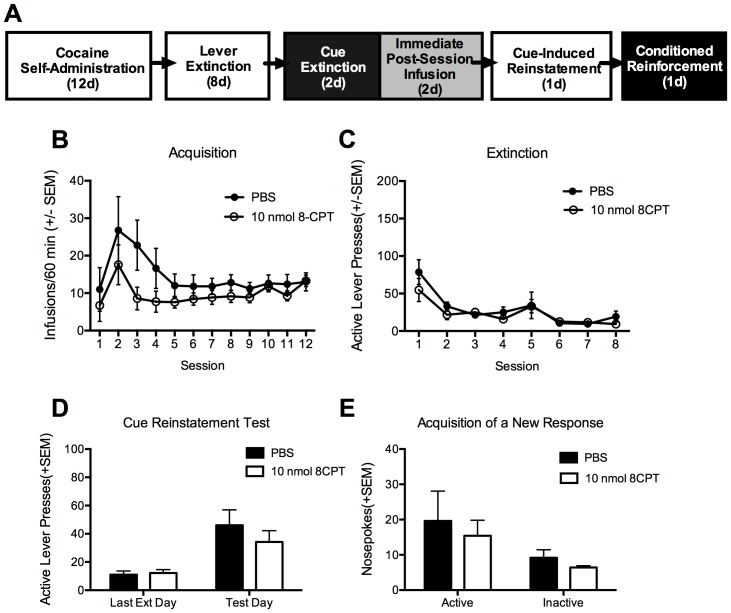
Epac activation by 8-CPT after Pavlovian extinction does not alter renewal of cocaine seeking or expression of extinction. A. Timeline of the experimental events. Black and white boxes are used to represent different contexts. B. Total number of cocaine infusions across the 12 acquisition sessions of cocaine self-administration. C. Total number of active lever presses on each day of extinction training. D. Total number of active lever presses on the last day of extinction compared to the cue-induced reinstatement test. E. Total number of active and inactive nosepokes on the acquisition of a new response test for conditioned reinforcement. All data are represented as the mean +SEM, PBS N = 5, 8-CPT N = 6.

In experiment 5, we determined if the apparent effect of Epac activation to inhibit drug memory reconsolidation could alternatively be occurring through an enhancement in Pavlovian extinction and reduction in renewal. In this experiment, rats were given the Epac activator 8-CPT immediately following each of two Pavlovian extinction sessions in a novel context that was identical to the reactivation context used in the experiments described above. Figure 5A illustrates the experimental timeline. Prior to manipulation, there were no statistical differences between groups on the number of infusions of cocaine acquired (Fig. 5B), active or inactive lever presses, or in active (Fig. 5C) or inactive lever presses during lever extinction training (all p’s>0.05). Following Pavlovian extinction manipulations, rats were tested for cue-induced reinstatement (renewal) in the original drug self-administration context where, as expected, there was a significant effect of day [F_(1,9)_ = 24.84, p<0.001], indicating a significant reinstatement effect, but there was no effect of treatment group or interaction (p’s >0.05), indicating that Epac activation after Pavlovian extinction training did not influence renewal of cocaine seeking (Fig. 5D). We expected animals to show renewal because extinction occurred in a different context, consistent with the methodology of the reconsolidation experiments, but because extinction is context-specific we predicted to see high levels of responding during the cue induced reinstatement test when the animals returned to the original training context. On the following day, rats were tested for their ability to retrieve the Pavlovian extinction memory in the extinction-training context, where we expected responding to be low, using the acquisition of a new response test for conditioned reinforcement. Analysis of these data indicated a significant effect of nose poke aperture (active vs. inactive) indicating that the animals did respond more for the conditioned cue than an inactive nosepoke [F_(1,10)_ = 6.86, p = 0.026], though there was much less responding compared to the reactivation control groups (see Fig. 3D). However, there was no effect of treatment group or interaction (p’s >0.05), indicating that Epac activation post-Pavlovian extinction training did not impair or enhance the consolidation or expression of extinction learning (Fig. 5E).

### Histology

**Figure 6:**
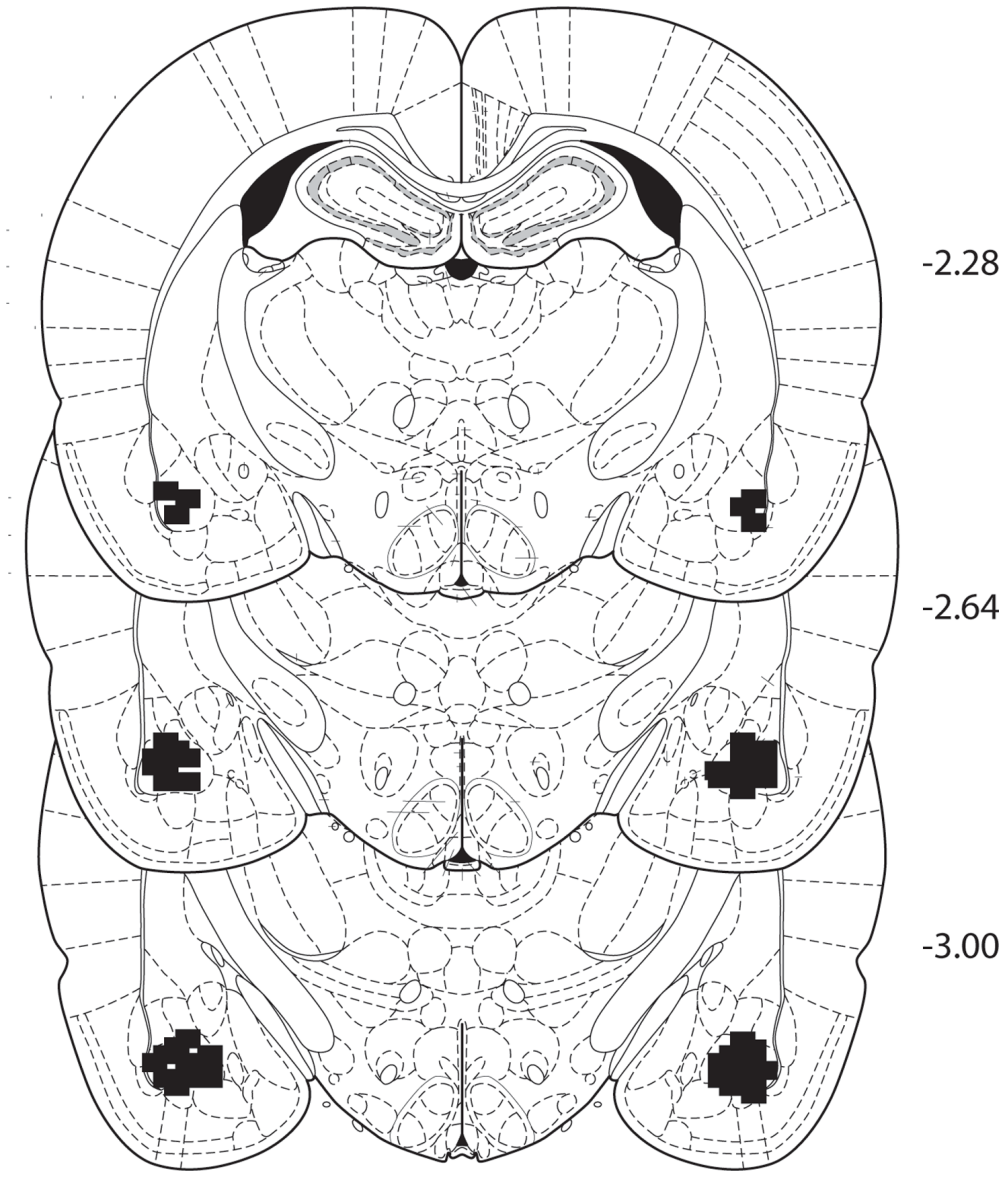
Diagram showing the region where all infusions were located. The infusion sites were marked based on Paxinos and Watson (2005). The numbers on each plate indicate the anterior-posterior position relative to bregma (mm).

For all experiments, we performed histological analysis of the location of the tips of all microinfusion cannulae and removed any animals that had placements outside the borders of the BLA from analysis. Figure 6 illustrates the location of the cannulae placements.

## Discussion

The present study demonstrates that intra-BLA infusion of the selective Epac activator 8-CPT immediately following reactivation of a drug-cue memory resulted in diminished effects on subsequent cue-induced reinstatement of cocaine-seeking behavior. These data suggest that activation of Epac inhibited memory reconsolidation and thereby mnemonic function associated with the motivational impact of drug-associated stimuli on behavior. In addition, activation of Epac did not alter Pavlovian extinction of cocaine cue memories, indicating that the effects observed were not due to an enhancement of extinction learning. The effects of Epac activation were temporally sensitive, such that no effect was found if the activator was infused 3 hrs after the reactivation session. These observations closely resemble previous reports that immediate post-reactivation inhibition of BLA PKA activity disrupted reconsolidation of appetitive (cocaine) and aversive (foot shock) cue memories [20], [21]. In addition, a novel finding in the present study is that when the PKA activator 6-BNZ was simultaneously administered with 8-CPT, the disruptive effects of 8-CPT on cue memory reconsolidation were blocked. In this experiment, PKA activation alone was not sufficient to modulate memory reconsolidation or subsequent cue-induced reinstatement. These data strongly suggest that these two cAMP-activated molecular signaling cascades have opposing effects when activated. However, under physiological conditions, we believe that PKA is preferentially activated during reconsolidation and that Epac is either less activated or activated at a later time point. Future experiments should determine the precise relationship between cAMP-induced activation of PKA versus Epac *in vivo*. However, when normal signaling is disrupted by activating Epac immediately after memory reactivation, then Epac-mediated Rap1 activation and dendritic spine destabilization [31] inhibits memory reconsolidation. Activation of PKA is able to overcome this disruption either by out competing Epac to maintain the memory or through bypassing a potential direct inhibitory effect of Epac on PKA function. These findings strongly suggest the possibility that the disruptive effects of Epac activation on memory reconsolidation are at least partly mediated by inhibition of PKA or PKA-activated substrates. However, interactions with other signaling cascades cannot be completely ruled out. Finally, while there was no significant effect of 6-BNZ alone to enhance memory reconsolidation as was observed for conditioned fear [20], there was a tendency to increase reinstatement. Thus, it may be that 6-BNZ can enhance memory strength for cocaine as well, but animals had reached a ceiling level of responding.

The post-reactivation manipulations we used in the present study are aimed specifically at the reconsolidation process [3], [7]. It is possible that the disruptions caused by Epac activation are mediated by impaired re-storage of the original memories, or a subsequent difficulty in retrieval, or both (see [7], [32]–[36]). There is evidence that BLA manipulations at the time of reactivation can endure up to 50 d, arguing for alterations in memory re-storage, which is expected to have long-term instead of transient effects [17]–[19]. It is notable that in the present study both reactivation and pharmacological manipulations occurred away from the context in which cocaine self-administration and reinstatement testing were conducted. Thus, the subsequent disruptions in cue-induced reinstatement suggest that manipulations of memory reconsolidation can be context independent, in contrast to exposure/extinction therapy, which generally cannot eliminate a return of the extinguished stimulus memory upon a change in the physical or temporal context (e.g., [2]–[5]. Therefore, manipulations of memory reconsolidation might provide a potentially more efficacious therapeutic strategy, and Epac a novel target.

Previous studies in our laboratory have shown that amygdalar PKA alone plays a critical role in memory reconsolidation in both appetitive (cocaine self-administration) and aversive (auditory fear conditioning) stimulus memories [7], [21]. It is well known that PKA exerts its mnemonic effects through various cellular processes including the activation of transcription factors, such as CREB and Zif268 (e.g., [7], [37], [38]), and regulation of synaptic activity, such as phosphorylation of GluR1 receptor subunits [39]. On the other hand, to date most studies on the biological functions of Epac have focused on systems other than the central nervous system (for reviews, see [40], [41]). Nonetheless, studies from neural tissues have begun to elucidate potential Epac-mediated molecular/cellular mechanisms that could have contributed to the observed disruption of drug-cue memory reconsolidation. For instance, Epac activates the neuroplasticity-related G protein Rap1 both in vitro [23] and in vivo [39]. In its active form, Rap1 contributes to formation of thin spines and AMPA receptor endocytosis, which could prevent memory restabilization. Also, it has been shown that upon activation, one of the isoforms of Epac (Epac2) induces consequences that could negatively regulate neuroplasticity, including spine shrinkage, removal of synaptic GluR2/3-containing AMPA receptors, and a decline in excitatory transmission [31]. Thus, Epac appears to be involved in the dynamic synapse remodeling that is related to both normal and pathological neuroplasticity, including memory formation. Finally, in various *in vitro* preparations such as hippocampal long-term potentiation studies, Epac-mediated signaling is involved in changes in synaptic transmission as well as excitability [42], [43].

To date, very few functional studies have directly examined interactions between cAMP targets and there have been no such published reports within the realm of PKA and Epac interaction underlying drug-cue memory processes. At the behavioral level, a couple of studies have demonstrated that PKA and Epac act synergistically to mediate hippocampus-dependent memory processes. For example, Epac activation has been shown to be sufficient to rescue retrieval deficits in norepinephrine/epinephrine -deficient *Dbh* ^−/−^ mice. The effect can be enhanced by co-administration of a PKA activator or blocked by a PKA inhibitor [44]. Similarly, in a hippocampus-dependent memory consolidation task, co-infusion of the Epac activator with a PKA inhibitor left fear memories intact while infusion of the PKA inhibitor alone impaired memory formation [39]. However, in the present study Epac and PKA appear to act antagonistically within the BLA because the disruptive effect of Epac activation on memory reconsolidation was blocked by simultaneous PKA activation. The behavioral effects of Epac and PKA co-administration may depend on the specific conditions such as aversive versus appetitive conditioning, or retrieval versus consolidation versus reconsolidation, or the brain region studied. The time course of PKA and Epac activation might also be an important factor that determines how the two molecules interact with each other. We have begun to examine the exact nature of the “crosstalk” between Epac and PKA signaling at the molecular and cellular level. These experiments confirm that either pharmacological activation or over-expression of Epac reduces PKA-dependent phosphorylation of the GluR1-subunit *in vitro* and *in vivo* ([45]; Angus Nairn, personal communication), suggesting that following cAMP-dependent activation of PKA and Epac, the two molecules may also directly interact with each other or downstream targets of Epac can interfere with PKA signaling. Because of the potentially more selective actions of Epac, pharmacological compounds that target the activity of small GTPases or their regulators, may have advantages therapeutically over manipulations of targets such as PKA and Zif268 that are likely to have overly broad effects.

Taken together, the present study demonstrates that BLA Epac activation immediately following reactivation of drug-cue memories can disrupt the ability of that cue to support subsequent cocaine-seeking behavior. This effect is independent of the context, is dose-dependent, occurs in a time-restricted manner, can be blocked by simultaneous PKA activation, and is not due to altered extinction learning. These observations extend what has been reported previously by manipulating BLA PKA activity, suggesting Epac as an additional target through which cAMP signaling can exert an impact on cognitive and motivational functions. Moreover, the nature of reconsolidation-targeted manipulations may suggest an especially efficacious avenue of therapies aimed at the elimination of highly intrusive memories that often result in debilitating psychiatric disorders like drug-craving and relapse in addiction as well as traumatic flashbacks in posttraumatic stress disorder [7], [46], [47].
